# Trends and predictors of non‐communicable disease multimorbidity among adults living with HIV and receiving antiretroviral therapy in Brazil

**DOI:** 10.1002/jia2.25233

**Published:** 2019-01-29

**Authors:** Jessica L Castilho, Maria M Escuder, Valdiléa Veloso, Jackeline O Gomes, Karu Jayathilake, Sayonara Ribeiro, Rosa A Souza, Maria L Ikeda, Paulo R de Alencastro, Unai Tupinanbas, Carlos Brites, Catherine C McGowan, Alexandre Grangeiro, Beatriz Grinsztejn

**Affiliations:** ^1^ Division of Infectious Diseases Vanderbilt University Medical Center Nashville TN USA; ^2^ São Paulo State Department of Health Institute of Health São Paulo Brazil; ^3^ National Institute of Infectology – Evandro Chagas Oswaldo Cruz Foundation Rio de Janeiro Brazil; ^4^ São Paulo State Department of Health AIDS Reference and Training Center São Paulo Brazil; ^5^ School of Health University do Vale do Rio dos Sinos Porto Alegre Brazil; ^6^ Care and Treatment Clinic of the Partenon Sanatorium Rio Grande do Sul State Department of Health Porto Alegre Brazil; ^7^ Medical School Federal University of Minas Gerais Belo Horizonte Brazil; ^8^ Edgar Santos University Hospital Complex Federal University of Bahia Salvador Brazil; ^9^ Department of Preventive Medicine University of São Paulo School of Medicine São Paulo Brazil

**Keywords:** HIV, ART, multimorbidity, non‐communicable diseases, ageing, Brazil

## Abstract

**Introduction:**

People living with HIV (PLHIV) on antiretroviral therapy (ART) experience high rates of non‐communicable diseases (NCDs). These co‐morbidities often accumulate and older adults may suffer from multimorbidity. Multimorbidity has been associated with loss of quality of life, polypharmacy, and increased risk of frailty and mortality. Little is known of the trends or predictors NCD multimorbidity in PLHIV in low‐ and middle‐income countries.

**Methods:**

We examined NCD multimorbidity in adult PLHIV initiating ART between 2003 and 2014 using a multi‐site, observational cohort in Brazil. NCDs included cardiovascular artery disease, hyperlipidemia (HLD), diabetes, chronic kidney disease, cirrhosis, osteoporosis, osteonecrosis, venous thromboembolism and non‐AIDS‐defining cancers. Multimorbidity was defined as the incident accumulation of two or more unique NCDs. We used Poisson regression to examine trends and Cox proportional hazard models to examine predictors of multimorbidity.

**Results:**

Of the 6206 adults, 332 (5%) developed multimorbidity during the study period. Parallel to the ageing of the cohort, the prevalence of multimorbidity rose from 3% to 11% during the study period. Older age, female sex (adjusted hazard ratio (aHR) = 1.30 (95% confidence interval (CI) 1.03 to 1.65)) and low CD4 nadir (<100 vs. ≥200 cells/mm^3^ aHR = 1.52 (95% CI: 1.15 to 2.01)) at cohort entry were significantly associated with increased risk of multimorbidity. Among patients with incident multimorbidity, the most common NCDs were HLD and diabetes; however, osteoporosis was also frequent in women (16 vs. 35% of men and women with multimorbidity respectively).

**Conclusions:**

Among adult PLHIV in Brazil, NCD multimorbidity increased from 2003 to 2014. Females and adults with low CD4 nadir were at increased risk in adjusted analyses. Further studies examining prevention, screening and management of NCDs in PLHIV in low‐ and middle‐income countries are needed.

## 1 Introduction

In countries with wide availability of antiretroviral therapy (ART), non‐communicable diseases (NCDs) increasingly account for morbidity and mortality among people living with HIV (PLHIV) 1, 2, 3. PLHIV are living longer and accumulating NCDs, resulting in multimorbidity, defined as two or more chronic NCDs 2, 4, 5, 6. In PLHIV , NCDs have been associated with decreased quality of life and physical function, dementia and mortality 7, 8, 9, 10, 11.

While much has been reported of NCDs in PLHIV in high‐income countries, understanding the burden of NCDs and other ageing‐related outcomes is of growing concern in low‐ and middle‐income countries 12, 13, 14. Brazil is an important country to study long‐term outcomes of PLHIV given its early availability of ART. Like many other middle‐income countries, the prevalence of a number of NCDs is increasing in the general population of Brazil 15, 16, 17, 18. Whether NCD trends among PLHIV mirror temporal trends has not been well described.

We sought to describe trends of NCD multimorbidity among adult PLHIV receiving ART in Brazil. We examined trends of individual NCDs and hypothesized that multimorbidity from NCDs would increase over time. We analysed patient characteristics associated with risk of multimorbidity and hypothesized that the risk would differ by demographic characteristics.

## 2 Methods

Coorte Brasil is a multi‐site observational cohort that was supported by the Brazilian Ministry of Health to examine outcomes of adult PLHIV initiating ART. The composition of the cohort has been previously described 19. The study abstracted routinely collected demographic, social, laboratory and clinical data from medical records and national databases of adults (ages ≥18 years) initiating ART between 2003 and 2014. In the first phase of Coorte Brasil, the Institutional Review Board (IRB) waived the requirement for written informed consent and requested the confidentiality of all data, which was ensured throughout the project. In the second phase, all participants provided written consent. Databases were harmonized, de‐identified and cleaned at the regional data centre in São Paulo.

Coorte Brasil partnered with the Caribbean, Central and South America network for HIV epidemiology (CCASAnet) to validate NCDs in a subset of cohort sites. The seven clinical sites included the Instituto Nacional de Infectologia – FIOCRUZ (Rio de Janeiro, RJ); AIDS Reference and Training Center (São Paulo, SP); São Paulo State Municipal Health Department – Santana (São Paulo, SP); São Paulo State Municipal Health Department – São Jose do Rio Preto (São Jose do Rio Preto, SP); Care and Treatment Clinic of the Partenon Sanatorium (Porto Alegre, RS); Federal University of Minas Gerais (Belo Horizonte, MG); and Edgar Santos University Hospital Complex (Salvador, BA). All NCDs were previously abstracted during the first phases of Coorte Brasil from medical records or were derived from collected laboratory values by the data‐coordinating centre. The NCD project validated the following previously collected incident and prevalent diagnoses: (1) coronary artery disease (including myocardial infarction and ischaemia), (2) cerebrovascular disease (cerebrovascular events and transient ischaemic attacks), (3) high‐grade hyperlipidemia (HLD, total cholesterol >300 mg/dL or low‐density lipoproteins >190 mg/dL, or triglycerides >750 mg/dL), (4) venous thromboembolism, (5) diabetes, (6) chronic kidney disease (serum creatinine ≥1.5 mg/dL for at least three months and including end stage renal disease), (7) cirrhosis, (8) osteonecrosis, (9) osteoporosis (osteoporosis, osteopenia and fragility fractures), and (10) all non‐AIDS‐defining cancers. NCDs were selected for their role in morbidity and mortality, reflection of end‐organ effects, reported association with HIV and/or ART, and feasibility of validation. Using a standardized protocol, researchers at each site reviewed medical charts of individual events for validation based upon physician documentation, objective evidence (such as pathology for cancer diagnoses, radiology for osteoporosis or laboratory results for metabolic outcomes), and/or prescription of medical treatment. Only diagnoses with strong evidence of support were kept for analyses. Sites additionally underwent data quality review by external auditors. This protocol was approved by the IRBs of the participating sites and Vanderbilt University.

This analysis examined multimorbidity related to NCDs following cohort entry (date of ART initiation). Multimorbidity was defined as two or more of the ten NCD diagnoses (above). NCDs were selected for the definition of multimorbidity given their clinical significance, potential morbidity and mortality, and validated endpoints. Diagnoses that were not collected nor validated were not included in the definition of multimorbidity. Each NCD diagnosis was treated as a chronic, non‐reversible and non‐repeating condition. We included all patients for calculation of trends over time. Time‐to‐event analyses of predictors of multimorbidity excluded those patients with prevalent multimorbidity at cohort entry. Patients with one NCD diagnosis at ART initiation were included in multimorbidity analyses. All patients were followed until last clinic visit, date of death or were censored at 31 December 2014, for those followed beyond 2014.

This study included demographic, clinical and laboratory data at ART initiation. CD4 cell count (CD4, cells/μL) and plasma HIV RNA (copies/mL) values were collected from the Brazilian national laboratory network. CD4 nadir was the lowest recorded CD4 prior to or at cohort entry. Baseline CD4 and HIV RNA were defined as the value closest to the date of ART initiation (no more than 180 days prior to or 30 days after).

We examined incidence trends of individual NCDs over the study period using Poisson regression including all persons in the cohort. We examined characteristics associated with multimorbidity risk by calculation of cumulative incidence curves and Cox proportional hazard models, after excluding patients with ≥2 NCDs at baseline. Cox models were stratified by clinical site to allow for differences in baseline hazards. Patients were censored once they developed ≥2 NCDs. Adjusted Cox models included sex, age at cohort entry, race, education, tobacco and alcohol use, hepatitis C virus infection, calendar year, CD4 nadir, HIV RNA and initial antiretroviral regimen. As certain NCDs increase the risk of others (such as diabetes and cardiovascular disease), adjusted models also included a covariate for the presence of one NCD at baseline. Variables included in the adjusted analyses were selected *a priori* based upon plausible biologic pathways. We examined NCD diagnoses among patients with incident multimorbidity using chi‐square tests. As a sensitivity analysis to evaluate consistency across NCD outcomes and recognizing the limitations of multimorbidity definition, we examined patient characteristics associated with the development of the first of any NCD using Cox proportional hazard models, excluding those patients with any prevalent NCDs at baseline.

Statistical analyses and figures were performed using Stata 12.1 (Stata Corporation, College Station, Texas, USA). All *p* values are two‐sided.

## 3 Results

**Figure 1:**
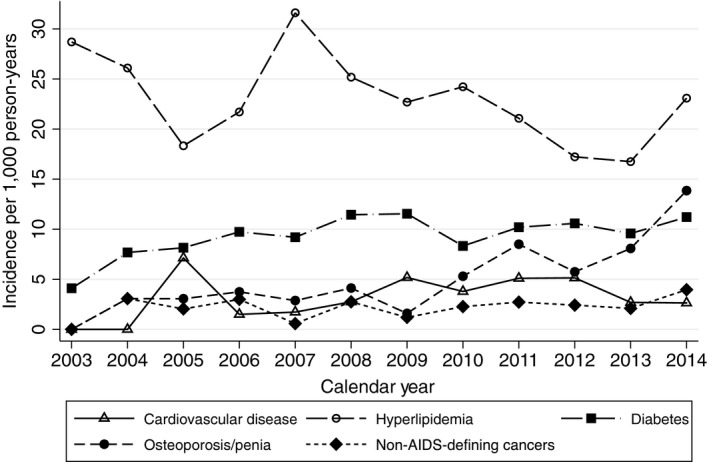
Incidence of most frequent NCDs observed during study period

**Figure 2:**
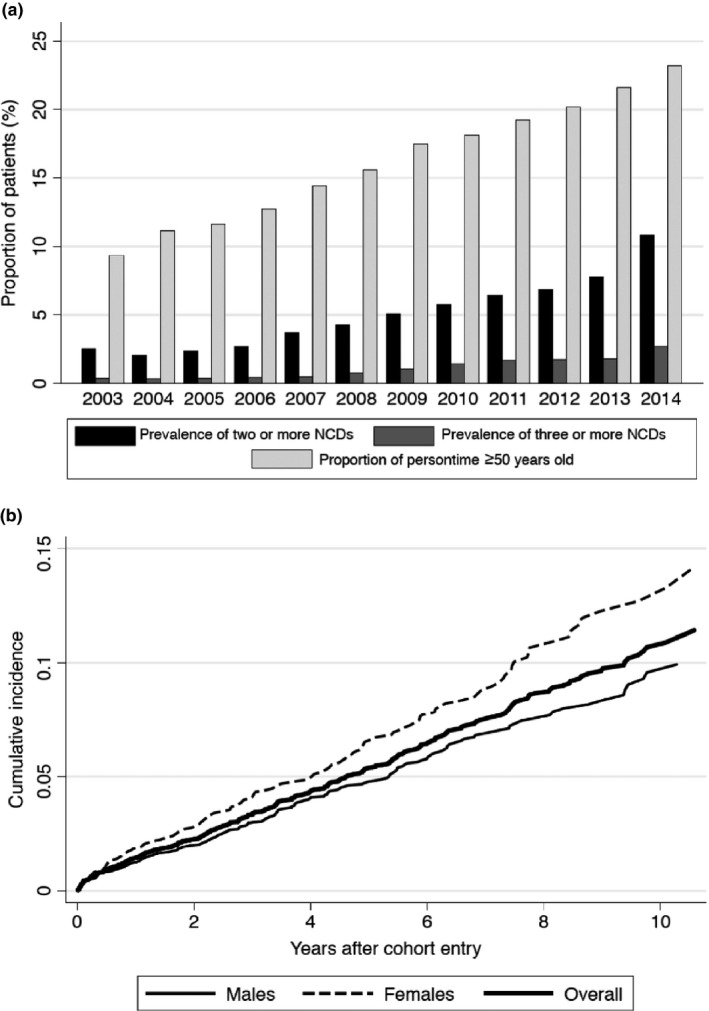
(a) Prevalence of multimorbidity and ageing of cohort. (b) Cumulative incidence of multimorbidity

There were a total of 6206 patients in the cohort, whom contributed 24,003 person‐years of observation. The baseline characteristics of the cohort are shown in Table 1. There were a total of 1158 incident NCD diagnoses among all patients. The most frequent diagnoses were high‐grade HLD (n = 515), diabetes (n = 233), and osteoporosis/osteopenia (n = 134). There were 51 incident coronary artery disease diagnoses, 53 non‐AIDS‐defining cancer diagnoses (the most frequent of which were non‐melanoma skin cancers (n = 11), lung cancer (n = 6), and Hodgkin's disease (n = 5)), 44 venous thromboembolism events, 44 chronic kidney disease events, 37 cirrhosis diagnoses, 35 cerebrovascular disease diagnoses and 12 osteonecrosis diagnoses. Figure 1 shows rates of the most frequent NCDs (coronary artery disease and cerebrovascular disease are combined in cardiovascular disease). The incidence of cardiovascular disease, non‐AIDS‐defining cancers, diabetes, kidney disease and cirrhosis remained statistically unchanged during the study period (*p* trend >0.05 for all). The rate of HLD decreased (*p *=* *0.03) while the rate of osteoporosis/osteopenia increased (*p *<* *0.001). The prevalence of multimorbidity steadily increased during the study period (*p *<* *0.001), paralleled by the increasing proportion of patients ≥50 years in the cohort (Figure 2a).

**Table 1:** Patient characteristics by sex

	Males (N = 4159)	Females (N = 2047)	Total (N = 6206)	*P* value^a^
Age at cohort entry in years, median (IQR)	36.5 (30.2 to 43.8)	37.5 (30.3 to 45.4)	36.9 (30.3 to 44.3)	0.007
HIV transmission risk factor, n (%)				
Heterosexual	1574 (38)	1670 (82)	3244 (52)	<0.001
Men who have sex with men	1865 (45)	0 (0)	1865 (30)	
Injection drug use	164 (4)	40 (2)	204 (3)	
Other/Unknown/Missing	556 (14)	337 (16)	893 (14)	
Race				
White	2324 (56)	998 (49)	3322 (54)	<0.001
Black or Mixed black	1624 (39)	955 (47)	2579 (42)	
Other/Unknown/Missing	211 (5)	94 (5)	305 (5)	
Education				
<9 years	1620 (39)	1155 (56)	2775 (45)	<0.001
≥9 years	2014 (48)	613 (30)	2627 (42)	
Unknown or missing	525 (13)	279 (14)	804 (13)	
Ever tobacco use				
Yes	910 (22)	599 (29)	1509 (24)	<0.001
No	2195 (53)	1022 (50)	3217 (52)	
Missing	1054 (25)	426 (21)	1480 (24)	
Ever alcohol use				
Yes	737 (18)	655 (32)	1392 (22)	<0.001
No	2012 (48)	755 (37)	2767 (45)	
Missing	1410 (34)	637 (31)	2047 (33)	
Years in clinic before cohort entry	0.28 (0.09 to 2.03)	0.50 (0.09 to 2.61)	0.34 (0.09 to 2.19)	<0.001
AIDS‐defining illness before cohort entry	1653 (40)	656 (32)	2309 (37)	<0.001
Tuberculosis before cohort entry	685 (16)	253 (12)	938 (15)	<0.001
Hepatitis C virus infection^b^	354 (9)	174 (9)	528 (9)	0.988
Chronic hepatitis B virus infection^c^	159 (4)	29 (1)	188 (3)	<0.001
CD4 cell count nadir at cohort entry (cells/μL)^d^	199 (82 to 290)	222 (115 to 295)	208 (91 to 292)	<0.001
CD4 cell count at cohort entry (cells/μL)^d^	234 (107 to 333)	248 (140 to 344)	238 (118 to 337)	<0.001
Log10 HIV RNA (copies/mL) at cohort entry	4.76 (4.16 to 5.22)	4.47 (3.79 to 5.07)	4.69 (4.03 to 5.18)	<0.001
Undetectable HIV RNA at baseline				
No	3216 (77)	1539 (75)	4755 (77)	0.121
Yes	402 (10)	228 (11)	630 (12)	
Missing baseline HIV RNA	541 (13)	280 (14)	821 (13)	
Year of ART initiation	2009 (2006 to 2011)	2008 (2006 to 2011)	2009 (2006 to 2011)	<0.001
ART regimen				
NNRTI‐based	3031 (73)	1244 (61)	4275 (69)	<0.001
PI‐based	1028 (25)	762 (37)	1790 (29)	
Other	100 (2)	41 (2)	141 (2)	
Nucleoside reverse transcriptase inhibitor in regimen				
Zidovudine	2684 (65)	1446 (71)	4130 (67)	<0.001
Tenofovir	1278 (31)	479 (23)	1757 (28)	
Other or none	197 (5)	122 (6)	319 (5)	
Follow‐up years after cohort entry	3.7 (1.8 to 6.5)	4.5 (2.0 to 7.0)	3.9 (1.9 to 6.8)	<0.001
Prevalent NCDs at cohort entry				
None	3923 (94)	1899 (93)	5822 (94)	0.017
One	179 (4)	122 (6)	301 (5)	
Two or more	58 (1)	27 (1)	85 (1)	
Prevalent NCDs at cohort entry				
Coronary artery disease	17 (0)	15 (1)	32 (1)	0.094
Cerebrovascular disease	13 (0)	12 (1)	25 (0)	0.110
High grade hyperlipidemia	104 (3)	51 (3)	155 (3)	0.983
Venous thromboembolism	5 (0)	5 (0)	10 (0)	0.252
Diabetes	103 (3)	58 (3)	161 (3)	0.406
Chronic kidney disease	24 (1)	11 (1)	35 (1)	0.844
Cirrhosis	14 (0)	7 (0)	21 (0)	0.973
Osteoporosis/osteopenia	4 (0)	14 (1)	18 (0)	<0.001
Non‐AIDS cancer	14 (0)	4 (0)	18 (0)	0.331
Death during follow‐up	412 (10)	181 (9)	593 (10)	0.180
Loss to follow‐up	1160 (28)	657 (32)	1817 (29)	0.001
Clinical Site (city of location)				
Rio de Janeiro	1591 (36)	631 (31)	2222 (35)	<0.001
Porto Alegre	706 (17)	655 (32)	1361 (22)	
São Paulo (CRT)	784 (19)	185 (9)	969 (16)	
Belo Horizonte	363 (9)	176 (9)	539 (9)	
São Jose do Rio Preto	253 (6)	162 (8)	415 (7)	
Salvador	286 (7)	145 (7)	431 (7)	
São Paulo (Santana)	176 (4)	93 (5)	269 (4)	

ART, antiretroviral therapy; ARV, antiretroviral; CRT, AIDS Reference and Treatment Center; IQR, interquartile range; NCD, non‐communicable disease; NNRTI, non‐nucleoside reverse transcriptase inhibitor; PI, protease inhibitor. ^a^ *p* value results of Wilcoxon ranksum test of continuous variables and chi‐square test of categorical variables. ^b^Hepatitis C infection defined by positive anti‐hepatitis C virus antibody test at any point. ^c^Chronic hepatitis B infection defined by positive hepatitis B surface antigen detected at any point. ^d^426 patients (7%) with missing CD4 cell count nadir data. 429 patients (7%) with missing CD4 cell count data at cohort entry.

After excluding 85 patients with ≥2 NCDs at ART initiation, 332 of the 6121 remaining patients developed multimorbidity during follow‐up. Cumulative incidence of multimorbidity is shown in Figure 2b. Table 2 reports the results of the Cox models for predictors of multimorbidity. The strongest predictor was the presence of one prevalent NCD. Of the 332 patients with multimorbidity, 123 had one NCD at baseline. In adjusted models, older age, female sex, missing baseline HIV RNA and low CD4 nadir remained statistically associated with increased risk of multimorbidity. We found no meaningful associations between race, education, hepatitis C virus infection, calendar year, or specific antiretrovirals and multimorbidity risk. Adjusted models were repeated stratifying by sex and results were similar, statistical tests of interactions between sex and other covariates were statistically non‐significant (results not shown).

**Table 2:** Unadjusted and adjusted Cox proportional hazard models^a^ for NCD multimorbidity

	HR [95% CI]	*p* value	aHR [95% CI]	*p* value
Age at cohort entry				
<30 years	(Reference)		(Reference)	
30 to 39 years	1.56 [0.95 to 2.55]	0.078	1.38 [0.84 to 2.26]	0.207
40 to 49 years	3.58 [2.24 to 5.71]	<0.001	2.81 [1.75 to 4.50]	<0.001
≥50 years	11.39 [7.19 to 18.05]	<0.001	6.73 [4.19 to 10.8]	<0.001
Female sex	1.53 [1.22 to 1.91]	<0.001	1.30 [1.03 to 1.65]	0.027
HIV transmission risk factor				
Heterosexual	(Reference)			
Men who have sex with men	0.62 [0.48 to 0.81]	<0.001		
Injection drug use	0.80 [0.41 to 1.58]	0.524		
Other	1.67 [0.74 to 3.76]	0.219		
UNKNOWN OR MISSING	1.03 [0.71 to 1.50]	0.869		
Race				
White	(Reference)		(Reference)	
Mixed black or black	1.08 [0.86 to 1.35]	0.514	1.06 [0.84 to 1.35]	0.609
Other/Missing	1.04 [0.55 to 1.96]	0.898	0.81 [0.42 to 1.54]	0.521
Education				
≥9 years	(Reference)		(Reference)	
<9 years	1.45 [1.14 to 1.84]	0.003	1.16 [0.89 to 1.51]	0.267
Unknown or Missing	1.87 [1.16 to 3.02]	0.010	1.00 [0.62 to 1.61]	0.991
Ever tobacco use				
Yes	(Reference)		(Reference)	
No	0.84 [0.60 to 1.19]	0.331	0.80 [0.55 to 1.16]	0.267
Unknown/missing	0.67 [0.51 to 0.89]	0.005	0.80 [0.58 to 1.09]	0.991
Ever alcohol use				
Yes	(Reference)		(Reference)	
No	0.79 [0.53 to 1.16]	0.225	1.02 [0.68 to 1.53]	0.936
Unknown/missing	0.75 [0.57 to 0.99]	0.041	1.02 [0.75 to 1.39]	0.903
Years in clinic before ART initiation	1.00 [0.99 to 1.00]	0.910		
AIDS‐defining illness before cohort entry	1.11 [0.89 to 1.39]	0.336		
Tuberculosis before cohort entry	1.13 [0.86 to 1.50]	0.364		
Hepatitis C virus infection^b^	1.52 [1.07 to 2.16]	0.018	0.97 [0.68 to 1.39]	0.887
Chronic hepatitis B virus infection^c^	1.33 [0.76 to 2.34]	0.309		
Year of cohort entry	1.01 [0.97 to 1.06]	0.580	1.04 [0.99 to 1.10]	0.110
CD4 cell count nadir at cohort entry (cells/mm^3^)				
≥200	(Reference)		(Reference)	
100 to 199	1.38 [1.02 to 1.83]	0.035	1.47 [1.09 to 1.98]	0.012
<100	1.55 [1.19 to 2.01]	0.001	1.52 [1.15 to 2.01]	0.003
Missing	1.30 [0.85 to 1.99]	0.221	1.11 [0.64 to 1.93]	0.699
CD4 cell count at cohort entry (cells/mm^3^)				
<100	1.39 [1.03 to 1.93]	0.031		
100 to 199	1.41 [1.03 to 1.93]	0.031		
200 to 349	(Reference)			
≥350	1.15 [0.83 to 1.59]	0.385		
Missing	1.28 [0.84 to 1.97]	0.251		
Log10 HIV RNA at cohort entry	1.08 [0.93 to 1.25]	0.298		
HIV RNA at cohort entry				
Detectable	(Reference)		(Reference)	
Undetectable	1.18 [0.85 to 1.62]	0.325	1.11 [0.79 to 1.55]	0.565
Missing	1.29 [0.97 to 1.73]	0.084	1.82 [1.26 to 2.64]	0.002
Prevalent NCD at baseline	17.6 [14.0 to 22.1]	<0.001	13.2 [10.3 to 16.7]	<0.001
ART regimen				
NNRTI‐based	(Reference)		(Reference)	
PI‐based	0.99 [0.77 to 1.26]	0.922	1.12 [0.87 to 0.144]	0.377
Other	1.43 [0.76 to 2.70]	0.272	1.44 [0.74 to 2.79]	0.286
Nucleoside reverse transcriptase inhibitor				
Zidovudine	(Reference)		(Reference)	
Tenofovir	0.83 [0.62 to 1.10]	0.200	0.92 [0.67 to 1.25]	0.578
Other or none	1.51 [1.02 to 2.24]	0.040	1.18 [0.79 to 1.78]	0.420

aHR, adjusted hazard ratio; ART, antiretroviral therapy; CI, confidence interval; HR, hazard ratio; NCD, non‐communicable disease; NNRTI, non‐nucleoside reverse transciptase inhibitor; PI, protease inhibitor. ^a^Models stratified by clinical site in analyses. ^b^Hepatitis C infection defined by positive anti‐hepatitis C virus antibody test at any point. ^c^Chronic hepatitis B infection defined by positive hepatitis B surface antigen detected at any point.

We examined the frequencies of individual NCD diagnoses among patients with multimorbidity. The 332 patients with multimorbidity included 193 men and 139 women. For both men and women with multimorbidity, the most frequent NCD diagnosis was HLD which accounted for 40% of NCD diagnoses in men and 35% of NCD diagnoses in women. After HLD, diabetes was the next most frequent NCD for both men and women, accounting for approximately 25% of diagnoses in both men and women. However, the third most frequent NCD among women with multimorbidity was osteoporosis/osteopenia, accounting for 15% of all NCD in women and 7% of NCD diagnoses among men (*p *<* *0.001 for sex comparison).

Lastly, we examined patient characteristics associated with first NCD among those patients without any prevalent NCDs. In the adjusted model, older age at ART initiation (aHR for age ≥50 vs. <30 years = 6.61 (95% CI: 4.88 to 8.96)), and low CD4 nadir (aHR for <100 vs. ≥200 cells/mm^3^ = 1.36 (1.13 to 1.64)) were associated with increased risk of any incident NCD (results not shown).

## 4 Discussion

In this study of more than 6000 adult PLHIV initiating ART in Brazil, we found the prevalence of multimorbidity from NCDs steadily increased during the study period, and that older age, female sex, low CD4 nadir and missing HIV RNA were independently associated with increased risk of multimorbidity. Metabolic diagnoses of HLD and diabetes were the most frequent causes of multimorbidity.

While the rates of most of the NCDs remained statistically stable during the study period, the prevalence of NCD multimorbidity increased, concurrent with the ageing of the cohort population. By the end of the study period, more than 20% of all patients were over the age of 50 years and more than 10% had two or more NCD diagnoses. The growing burden of multimorbidity among ageing PLHIV has been observed globally where ART has been widely available 6. Studies from the United States and Europe have demonstrated that PLHIV experience higher rates of multimorbidity compared to their uninfected peers and that the burden of multimorbidity significantly increases at older ages 2, 4, 10, 20, 21. Consistent with other studies from Brazil, this study provides important evidence of this trend occurring in a middle‐income country where ART has been widely available since 1996 3, 22, 23.

The current study provides longitudinal data to examine NCD incidence trends in Brazil. Among the general population in Brazil, prevalence of a number of NCDs (particularly metabolic diseases) has increased over time 24. Metabolic disorders were the most frequent NCDs in our study. The rate of high‐grade HLD decreased during the study period, which may reflect the decreased use of lopinavir/ritonavir after 2009 (data not shown). In adjusted analyses, protease inhibitor use was not significantly associated with risk of multimorbidity, which may be a result of other contemporaneous changes in HLD risk related to diet or obesity or heterogeneity in multimorbidity outcomes. We observed a rise in the incidence of osteoporosis/osteopenia diagnoses after 2009. Osteoporosis screening with bone mineral densitometry was introduced as early as 2010 at some clinic sites and as late as 2013 in others, generally of patients over the age of 50 years and postmenopausal women. Screening was not uniform across sites and was often limited, particularly in public health settings. Additionally, an increasing proportion of patients in the cohort in later years received tenofovir (the median calendar year for patients who initiated zidovudine was 2007 vs. 2010 for those who initiated tenofovir), which has also been associated with osteoporosis risk 25, 26. The rise in osteoporosis incidence in our cohort most likely reflects introduction of even limited screening and ageing. These results highlight the need for additional screening for and study of bone‐related outcomes of ageing HIV‐positive adults in Brazil.

In our cohort, female sex was independently associated with increased risk of multimorbidity. Studies from high‐income countries have also found a higher burden of NCD co‐morbidity among women living with HIV compared to men 21. In the general population of Brazil, the rising burden from NCDs among women is a growing concern. A national health survey in 2013 highlighted a number of important NCD disparities in Brazil, including higher prevalence among women, older adults, and adults with low levels of education 27. Women in our study were followed up for longer periods of time, had lower risk of mortality (data not shown), and lower education than the men. However, multimorbidity risk associated with sex was independent of race, education, ART regimen or prevalent NCD at ART initiation in the adjusted Cox model.

Our study also found the association of low CD4 nadir associated with increased risk of NCD multimorbidity. Inflammation and immune activation are important contributors to the excess risk of cardiovascular disease, cancers, liver disease and other NCDs observed in PLHIV 28, 29, 30, 31. Low CD4 and CD4 nadir have been associated with persistent changes in the immune system (including inflammation) and development of NCDs 20, 32, 33, 34, 35. The median CD4 at ART initiation of <250 cells/μL in our cohort underscores the importance of ongoing efforts towards early HIV diagnosis and treatment to improve health outcomes, including those associated with ageing.

Our study has important strengths to highlight. This multi‐site study draws from a diverse clinical population in Brazil. The comprehensive data collected in Coorte Brasil was strengthened through additional standardized validation of NCDs. Our study also leveraged the Brazilian national systems of HIV laboratory data and death registry. However, we found a missing HIV RNA at cohort entry was associated with risk of multimorbidity, which may reflect those patients who were very ill at baseline, had less healthcare utilization, or whom obtained testing through private laboratories. Additionally, data on body mass index were not collected at study sites and behavioural data had a moderate degree of missingness. We examined key NCD diagnoses such as metabolic, cardiovascular, liver, renal, and cancer diagnoses that have been of particular focus in the HIV literature. However, there is no uniform list of conditions to include in defining multimorbidity and we did not include all possible NCDs such as pulmonary diseases (which were not collected in Coorte Brasil) or mental health disorders (which are inherently difficult to validate in observational research). Other chronic conditions contribute to multimorbidity in PLHIV and their exclusion from our study may have affected our results. Also, our results demonstrated that prevalent NCD at ART initiation was strongly associated with risk of multimorbidity, however this was limited by small numbers to further stratify on baseline NCD diagnosis to better understand how specific NCDs contribute to risk of future multimorbidity. Lastly, screening for a number of NCDs remains limited in Brazil and our results likely underestimate the overall burden of multimorbidity.

## 5 Conclusions

In conclusion, multimorbidity from NCDs is increasing among Brazilian PLHIV on ART. Our findings highlight the importance of metabolic disorders as leading causes of NCD morbidity. Future studies into the trends, outcomes, and policies in affecting NCDs in Brazil and other low‐ and middle‐income countries are needed.

## Competing interest

All authors of this study declare they have no competing interests.

## Authors’ contributions

M.M.E, V.V., J.O.G., S.R., R.A.S., M.L.R.I., P.R.A., U.T., C.B., A.G. and B.G. conducted the observational research and led data collection. J.L.C, S.R., K.J., J.O.G. and C.C.M. conducted data quality assessments. A.G., B.G., V.V., C.C.M., M.M.E. and J.L.C designed the study. J.L.C. and K.J. analysed the data. J.L.C, B.G., C.C.M, M.M.E and A.G. wrote the paper. All authors read and approve the final manuscript.
